# Clinical Stage III NSCLC Patients Treated with Neoadjuvant Therapy and Surgery: The Prognostic Role of Nodal Characteristics †

**DOI:** 10.3390/life12111753

**Published:** 2022-11-01

**Authors:** Marco Chiappetta, Diomira Tabacco, Amedeo Giuseppe Iaffaldano, Jessica Evangelista, Maria Teresa Congedo, Carolina Sassorossi, Elisa Meacci, Ettore D’Argento, Emilio Bria, Emanuele Vita, Giampaolo Tortora, Luca Boldrini, Diepriye Charles-Davies, Mariangela Massaccesi, Antonella Martino, Ciro Mazzarella, Vincenzo Valentini, Stefano Margaritora, Filippo Lococo

**Affiliations:** 1 Università Cattolica del Sacro Cuore, 00135 Rome, Italy; 2 Thoracic Surgery, Fondazione Policlinico Universitario A. Gemelli IRCCS, LARGO A. Gemelli 8, 00135 Rome, Italy; 3 Medical Oncology, Dipartimento di Medicina e Chirurgia Traslazionale, Università Cattolica del Sacro Cuore, 00168 Rome, Italy; 4 Radiotherapy Unit, Fondazione Policlinico Universitario A. Gemelli IRCCS, LARGO A. Gemelli 8, 00135 Rome, Italy

**Keywords:** NSCLC, lymph nodes, adjuvant therapy, neoadjuvant therapy

## Abstract

BACKGROUND: The aim of this study is to analyze the prognostic factors in patients that underwent induction therapy and surgery for clinical stage III NSCLC. METHODS: Clinical and pathological characteristics of stage III NSCLC patients for N2 involvement that underwent neoadjuvant treatment (NAD) and surgery from 1/01/1998 to 31/12/2017 were collected and retrospectively analyzed. Tumor characteristics, yClinical, yPathological stage and lymph node characteristics were correlated to Overall Survival (OS). RESULTS: The analysis was conducted on 180 patients. Five-year OS (5YOS) was 50.9%. Univariable analysis results revealed old age (*p* = 0.003), clinical N2 post-NAD (*p* = 0.01), pneumonectomy (0.005), persistent pathological N2 (*p* = 0.039, HR 1.9, 95% CI 1.09–2.68) and adjuvant therapy absence (*p* = 0.049) as significant negative prognostic factors. Multivariable analysis confirmed pN0N1 (*p* = 0.02, HR 0.29, 95% CI 0.13–0.62) as a favorable independent prognostic factor and adjuvant therapy absence (*p* = 0.012, HR 2.61, 95% CI 1.23–5.50) as a negative prognostic factor. Patients with persistent N2 presented a 5YOS of 35.3% vs. 55.8% in pN0N1 patients. Regarding lymph node parameters, the lymph node ratio (NR) significantly correlated with OS: 5YOS of 67.6% in patients with NR < 50% vs. 29.5% in NR > 50% (*p* = 0.029). CONCLUSION: Clinical response aided the stratification of prognosis in patients that underwent multimodal treatment for stage III NSCLC. Adjuvant therapy seemed to be an important option in these patients, while node ratio was a strong prognosticator in patients with persistent nodal involvement.

## 1. Introduction

The therapeutic strategy for non-small cell lung cancer (NSCLC) patients with mediastinal nodal involvement remains one of the most debated topics in thoracic oncology. Indeed, different possible treatments are available according to the actual guidelines [1,2,3], consisting of upfront surgery, neoadjuvant (NAD) therapy, plus surgical resection or definitive radiochemotherapy. In particular, induction therapy is usually related to survival improvement when compared with upfront surgery. Therefore, this strategy is recommended for the treatment of clinical N2 patients [4]. 

The clinical rationale of induction therapy gives the possibility to reduce tumor dimensions and nodal involvement with consequent downstaging, prognosis improvement and to also reduce the distant tumor spread, thereby improving survival [5,6,7]. However, in the past years different studies have investigated the possible role of NAD in NSCLC patients with inconclusive results, especially due to nonhomogeneous patient selection or the high number of surgery-related deaths in the surgical group [8,9]. In more detail, Albain et al. [9], in their randomized controlled trial, reported a better progression for survival in the NAD plus surgery group vs. the definitive radiochemotherapy group, while no differences were present considering overall survival, probably due to the higher number of postoperative deaths after pneumonectomy. For this reason, several fundamental points, such as patient selection, are yet to be evaluated and clarified. It will be essential to identify which preintervention parameters are capable of significantly predicting prognosis with the aim of appropriately selecting the patients to be referred for surgery or to be consolidated with an exclusive radiochemotherapy treatment. Although some parameters, such as the presence of limited nodal involvement, tumor shrinkage or lobectomy, have been proposed as favorable factors for inductive treatment followed by surgery, clear evidence is still missing [1,3]. However, different studies proposed potential prognostic factors in patients that underwent the multimodal treatment approach, with the possibility of survival improvement in 5–6% of the selected patients [10]. More specifically, pathological complete response and N2 downstaging were found to be significant prognostic factors in these patients and the metabolic response was considered more reliable compared to morphological evaluation only [10,11]. In recent years, different neoadjuvant protocols, including immunotherapy or target agents, have been introduced with encouraging results in terms of major, complete and nodal pathological response with consequent significant survival benefits [12,13].

For these reasons, the possible presence of lymph node factors that can significantly change the outcomes of these patients, such as the lymph node involvement/response pattern, have not been thoroughly analyzed. However, recent studies have started considering more deeply the role of nodal characteristics after NAD for prognosis prediction [14,15]. Although the presence of persistent disease after NAD is considered as a potential contraindication for surgical resection, it is important to note that the term “persistent”, in this case, is linked to a heterogeneous group of patients with single or multistation involvement or concomitant N1 disease. Despite the fact that some factors, such as the number of resected nodes or the ratio between the number of metastatic and resected nodes (node ratio), can emerge as strong prognostic factors in patients that underwent lung surgery [16,17], a specific analysis of NAD patients is still missing. As a result, the nodal characteristics may be investigated more thoroughly and may be useful for prognosis stratification or for planning adjuvant treatments. Indeed, the role of adjuvant treatments is still not clear, even if recent evidence suggests that postoperative radiotherapy may not confer any survival advantage after complete resections or after adjuvant chemotherapy administration [1,18].

The aim of this study is:

− To assess prognostic factors in patients with NSCLC with mediastinal lymph node involvement who underwent neoadjuvant therapy and surgical treatment;
− To describe lymph node-related variables influencing survival

## 2. Materials and Methods

The data of patients with clinical N2 involvement that underwent neoadjuvant treatment and surgery in our thoracic surgery unit from 1 January 1998 to 31 December 2017 were retrospectively collected and analyzed. The inclusion and exclusion criteria are reported in Table 1 below:

Initial staging consisted of the brain, thorax and abdomen computed tomography (CT scan) with contrast and/or brain magnetic resonance imaging (MRI), when indicated. From 2009, 18-FDG Position Emission Tomography (PET) was performed for staging purposes. Preoperative diagnosis was obtained via bronchoscopy or transparietal fine needle aspiration [19], while N2 pathological confirmation was obtained via mediastinoscopy, mediastinotomy or, after the adoption, using EBUS and/or EUS. 

Neoadjuvant treatments were administered according to the following schemes:

− Neoadjuvant radiochemotherapy (NAD RCT): Two cycles of platinum-based doublet chemotherapy were administered concurrently with the standard conventionally fractionated thoracic radiation therapy (total dose of 50.4 Gy, with fractionation of 1.8 Gy/die) with a linear accelerator (LINAC), using a conformal or intensity modulated technique to the primary tumor and involved regional nodes. In detail, the irradiation field involved the positive and the closest nodal stations.

In some cases, according to a multidisciplinary evaluation, NAD RCT was performed after two cycles of induction platinum-based doublet chemotherapy were administered concurrently with “ultra-fractionated low dose” radiotherapy (40 cGy twice daily, days 1–2 and 8–9, every 21 days), using a conformal technique to the primary tumor, involved regional nodes and elective nodal stations (those adjacent to the involved ones) [20].

− NAD chemotherapy (NAD CT): four cycles of platinum-based chemotherapy were associated with a second chemotherapy drug (mainly paclitaxel, gemcitabine, pemetrexed)

Restaging after neoadjuvant therapy was performed by CT scan with the contrast of the brain, thorax and abdomen and/or FDG-PET re-evaluation, if available and indicated. In case of suspected persistence of mediastinal nodal disease, a multidisciplinary discussion was organized to decide the therapeutic iter: definitive radiochemotherapy, minimally invasive or invasive nodal restaging or surgery. 

The clinical response to neoadjuvant treatment was considered and compared to the pretreatment clinical stage, which was defined as follows in accordance with the RECIST criteria [21]:

Downstaging: transition from stage III to lower stage (I-II or full clinical response)

Mediastinal downstaging: response on mediastinal lymph nodes with clinical/pathological stage N0–N1.

Surgery was performed with the aim of obtaining a complete resection in every patient, avoiding the execution of pneumonectomy whenever possible. Systematic lymph node dissection according to ESTS guidelines was planned for all patients [22]. This procedure was not performed or changed with sampling, in case of the patient’s instable hemodynamic condition and presence of sticky nonresectable lymph nodes due to infective disease or as a result of the induction treatments that make lymphadenectomy a high-risk procedure with major complications. 

Clinical information, imaging and pathological reports were reviewed, and patients were restaged according to the 8th TNM edition [23], classifying the pathological response as follows:

Downstaging: transition from stage III to lower stage (I–II or full pathological response).

Mediastinal downstaging: response on mediastinal lymph nodes with clinical/pathological stage N0–N1.

Complete response: absence of viable cancer cells in the specimen and lymph nodes removed [7].

In some cases, with respect to the pathological stage, postoperative node stage and patients’ clinical condition, adjuvant treatments were administered. In particular, adjuvant chemotherapy consisted of platinum-based doublet chemotherapy, usually administered 4–8 weeks after surgery for a total number of four cycles. Adjuvant radiotherapy was administered after a multidisciplinary discussion, based on the therapy performed in the neoadjuvant setting, extracapsular nodal involvement or resection margins close to the tumor.

Follow-up was performed by clinical examination, blood tests, CT scan and PET when deemed necessary and patients were evaluated every six months for the first three years and annually for the following years.

### Statistical Analysis

A descriptive analysis, including clinical and demographic characteristics of the patients, was analyzed through the median and the range for continuous variables and the absolute value and relative frequencies for categorical variables.

Overall survival was calculated from the surgery date to the date of death by any cause. 

Cancer Specific Survival (CSS) was calculated from the surgery date to death by tumor progression.

Clinical patient factors, clinical and pathological tumor characteristics, nodal characteristics, such as number of resected nodes (#RN), number of metastatic lymph nodes (#MN) and node ratio (#MN/#RN: NR), were correlated to OS. Survival curves were calculated by the Kaplan–Meier product-limit method from the date of surgery until relapse or death. The log-rank test was used to assess differences between subgroups. Significance was defined at the *p* ≤ 0.05 level. The Hazard Ratio (HR) and the 95% confidence intervals (95% CI) were estimated using the Cox univariate model. A multivariate Cox proportional hazard model was developed using stepwise regression (forward selection) to compare the prognostic power of different factors. Enter limit and remove limit were *p* = 0.10 and *p* = 0.15, respectively. Variables for multivariable analysis were also chosen considering their clinical relevance. The assessment of interactions between significant investigation variables was taken into account when developing the multivariate model. Statistical evaluations were performed using SPSS (v. 21.0, SPSS Inc., Chicago, IL, USA)

## 3. Results

**Figure 1:**
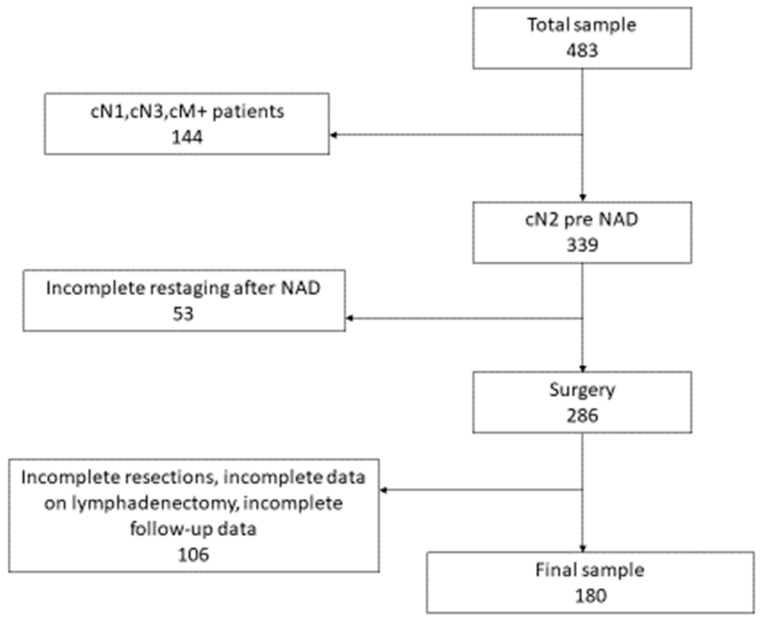
The flow chart of the study.

During the study period, 483 patients underwent NAD and surgical resection in our hospital and, according to the inclusion and exclusion criteria, the final analysis was conducted on 180 patients (Figure 1 flow chart). 

Clinical and pathological characteristics are reported in Table 2. In detail, clinical stage IIIA resulted in 148 (82.2%) patients and IIIB (T3-T4N2) in 38 (17.8%) patients. Moreover, adenocarcinoma was the most predominant histology.

After post-NAD re-evaluation, clinical N2 downstaging was observed in 137 (76.1%) patients, while for the remaining 43 patients, surgical indication was recommended after the multidisciplinary discussion (Table 3).

Pneumonectomy was performed in 40 patients and the mean number of resected lymph nodes was 8.5 ± 7.4, whereas postoperative and 30-day mortality were null. Seventy-seven patients died during follow-up and 65 out of the 77 recorded deaths were due to tumor-related causes.

Pathological analysis showed a complete response in 46 (25.5%) patients, downstaging in 137 (76.1%) patients and N2 downstaging in 151 cases, while a stable stage IIIA was present in 29 N2 and 14 T3N1patients. The mean #MN was 1.2 ± 2.9.

### Survival Outcome

Five years OS (5YOS) was 50.9%. In the univariable analysis, advanced age (*p* = 0.003), clinical N2 after NAD (*p* = 0.01), pneumonectomy (0.005), persistent pathological N2 (*p* = 0.039, HR 1.9, 95% CI 1.09–2.68) and absence of adjuvant therapy (*p* = 0.049) were regarded as significant negative prognostic factors (Table 4). Similarly, advanced age (*p* < 0.001), clinical N2 after NAD (*p* = 0.035) and pneumonectomy (0.003) were also considered as significant negative prognostic factors. From the analysis, the absence of adjuvant therapy raised the statistical significance (*p* = 0.057) (Supplemental Table S1).

**Figure 2:**
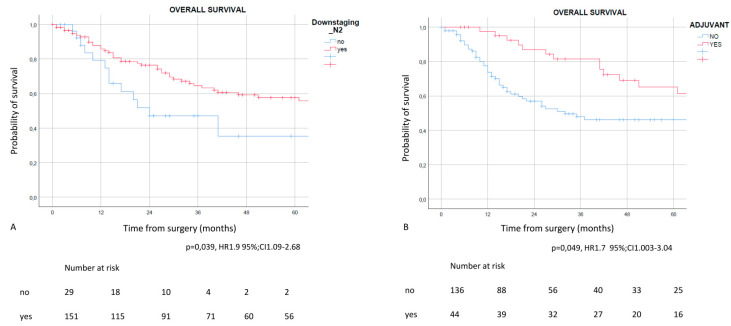
Overall survival in patients with pathological downstaging after NAD (**A**) and in patients that underwent adjuvant therapy (**B**). Patients presented a significant survival advantage when a N2 downstaging was present and when adjuvant therapy was administered.

On the other hand, multivariable analysis results (Table 5) confirmed post-treatment pathological N0N1 (*p* = 0.02, HR 0.29, 95% CI 0.13–0.62) as the favorable independent prognostic factor, while the absence of adjuvant therapy administration (5yOS 41.2% vs. 61.4%) in patients that underwent adjuvant therapy (*p* = 0.012, HR 2.61, 95% CI 1.23–5.50) (Figure 2), was the negative prognostic factor. Patients with pathologically persistent N2 presented a 5-year OS of 35.3% vs. 55.8% in patients that experienced nodal downstaging. Regarding CSS, multivariable analysis showed that advanced age (*p* = 0.001), clinical N2 persistence (*p* = 0.004) and adjuvant therapy administration (*p* = 0.027) were independent prognostic factors (Supplemental Table S1).

**Figure 3:**
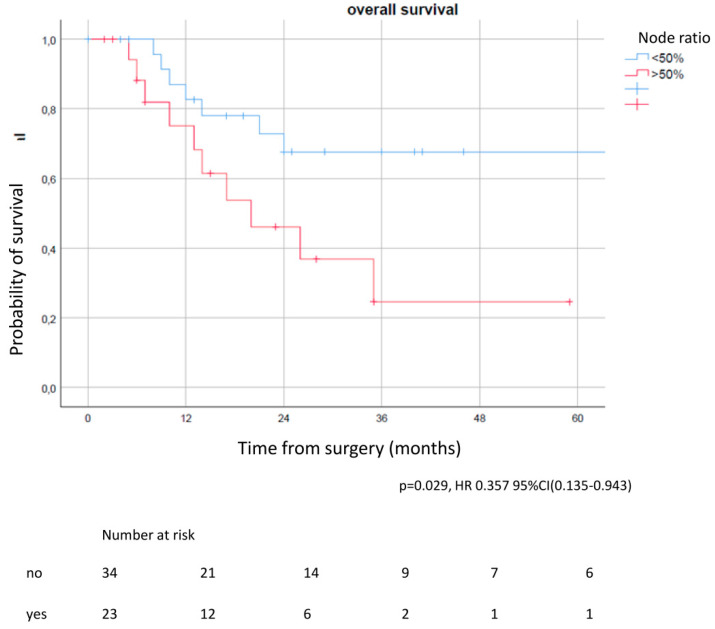
Overall survival according to lymph node ratio in patients with persistent nodal involvement. In patients with a node ratio < 50% the OS was significantly improved compared to patients with a node ratio > 50%.

Regarding lymph node parameters in patients with persistent nodal involvement, results from the univariable analysis revealed that the lymph node ratio (NR) significantly correlated with OS and CSS (5YOS of 67.6% in patients with NR < 50% vs. 29.5% in NR > 50%) (*p* = 0.029, Figure 3) (Table 6); 5YCSS of 84.7% in patients with NR < 50% vs. 27.3% in NR > 50% (*p* = 0.008, HR 0.170, 95% CI 0.046–0.632).

**Figure 4:**
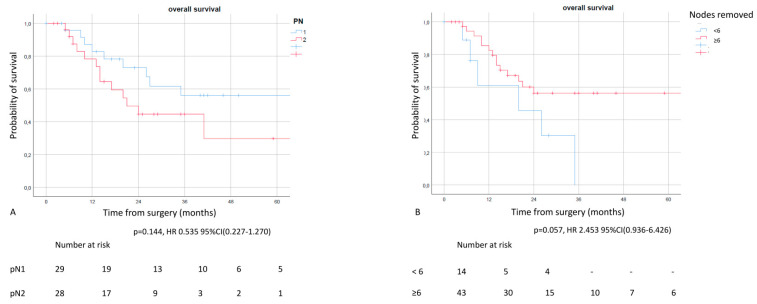
Overall survival according to pN (**A**) and number of resected mediastinal nodes (**B**) in patients with persistent nodal involvement. Although the result was not statistically significant, patients with N1 residual disease presented a better survival rate with clear curves separation compared to ypN2 patients. Similarly, patients with more than 6 resected nodes presented a better OS compared to patients with a limited number of resected nodes. These factors may be considered for prognosis and post-treatment evaluation in patients with persistent nodal involvement.

A difference was also observed between the number of resected lymph nodes and pathological N2 or N1 patients, even if it was not statistically significant: 5YOS of 56.1% in N1 vs. 29.7% in N2 (*p* = 0.144) and 5YOS of 0% in patients with #RN < 6 vs. 56.6% in patients with #RN ≥ 6 (*p* = 0.057) (Figure 4).

## 4. Discussion

Results from our study showed a fair 5-year survival rate of about 50% in patients that underwent induction treatment followed by surgery, reflecting the validity of this treatment in selected patients. 

Furthermore, we confirmed that lymph node response after NAD is a fundamental factor to consider for patient management in association with the type of planned surgical resection, which resulted in the worse prognosis in patients that underwent pneumonectomy [9]. 

In light of these two results, it is necessary to highlight that the management of this class of patients was successful because of the collaboration among members of a multidisciplinary team made up of: surgeons, oncologists, radiotherapists, pulmonologists, nuclear doctors, radiologists and pathologists, who ensured the correct referral of patients towards the appropriate strategy.

In addition, the evaluation of the response to treatment emerged as a key element in determining the prognosis of these patients. Re-evaluation exams, such as CT with contrast, PET or mini-invasive mediastinal restaging [11], were not only crucial to assess the response to treatment, but also to assess the type of intervention required for the patient. Indeed, the reduction of lymph node involvement and the size of the tumor can result in the resectability of the tumor and also increase the chances of obtaining an anatomically and oncological valid resection (lobectomy or bilobectomy), which is safer than pneumonectomy in terms of morbidity and mortality. Interestingly, new perspectives are developing in terms of the pathological response, with the inclusion of immunotherapy in neoadjuvant settings, with the hope that a good complete response rate may lead to improved survival [24].

For the sake of this study, we focused our attention on intraoperative prognostic factors, such as the kind of lymph node involvement, considering that data on lymphadenectomy in this setting were very limited. Although the indications do not differ from those used for patients referred directly for surgical treatment [2,22], lymphadenectomy in these patients can be a real challenge and it is rarely evaluated as a prognostic factor. 

Despite the addition of radiotherapy to the NAD schedule, morbidity rate did not seem to improve [25]. This could be as a result of modification of the irradiated areas, making the tissues sticky, difficult to recognize and, in some cases, tightly attached to the mediastinal structures in such a way that their removal was extremely risky. For this reason, it is not always possible to obtain a radical mediastinal lymphadenectomy. In fact, we observed that in patients with limited lymphadenectomy, survival was worse when compared to patients in whom a satisfactory number of lymph nodes was removed.

These data reflect the importance of lymph node and mediastinal downstaging in this class of patients [10,12] and the repercussions linked to the absence of lymph nodes analysis for the administration of adjuvant therapy in the nearest future.

In our study, adjuvant therapy emerged as an independent prognostic factor in these patients, suggesting its adoption whenever possible. Our results confirmed the results of Dr Scott et al. [26] that reported a significant survival advantage when adjuvant therapy was administered. However, it is important to note that this survival benefit was present only in patients with persistent nodal involvement. Therefore, it seems clear that adjuvant therapy administration requires the appropriate patient selection, which could be enhanced by understanding the nodal characteristics.

Furthermore, we also considered closely the type of lymph node parameters that may predict prognosis in ypN1–2 patients, knowing that the lymph node ratio aids stratification in these patients. 

Although the current TNM staging continues to classify patients according to the type of anatomical involvement (hilar N1 and mediastinal N2) and the combination of the nodal station involvement [27], it appears evident that other parameters allow for a more precise stratification of the prognosis in these patients. Among these parameters, the relationship between the number of metastatic lymph nodes and the number of lymph nodes removed is certainly one of the easiest to understand.

Moreover, the lymph node ratio makes it possible to have an initial impact on what may have been the spreading of the neoplasm, considering not only the number of lymph nodes involved, but also the extensivity of the lymph node assessment. In addition, some studies have shown that there is variability regarding the number of lymph nodes present within the lymph node chains, so the number of lymph nodes removed or metastatic lymph nodes could also be influenced by this anatomical variability [28].

This is the first time that the role of the lymph node ratio has been considered in this setting. Previously, other studies analyzed this parameter in a more or less heterogeneous population, but not with a specific focus on patients undergoing post-NAD surgery [16,17,29,30,31].

In this study, we tested a cut-off ratio of 50%. We observed that in patients with a ratio greater than 50%, the prognosis was extremely poor, with a 5-year CSS of about 30% vs. 80% in patients with a node ratio < 50%. Although it is a parameter that is difficult to predict preoperatively, it can be used to direct different patients towards suitable therapeutic solutions.

Indeed, the administration of adjuvant therapies in these patients remains controversial, and different efforts have been made to identify nodal parameters for patient selection. For instance, Dr Stamatis et al. [15] stratified patients according to the kind of N1/N2 involvement, showing a significantly worse prognosis in ypN1N2 patients and a good survival outcome in py-skip metastases. Similarly, Pataer et al. [14] evaluated the prognostic role of the nodal pathological response after NAD, thereby giving rise to new perspectives for specific patient selection for adjuvant therapy. In this context, the lymph node ratio could also be used to identify patients who could benefit from adjuvant treatments, such as postoperative radiotherapy in patients who still have margin for treatment, or they could be selected to continue consolidation chemotherapies postoperatively. In addition, it is possible that future indications for adjuvant therapy may be applied considering the type of persistent disease and nodal characteristics. However, this hypothesis needs to be verified in large ad hoc prospective studies. 

In this study we also analyzed the prognostic value of the number of lymph nodes removed, which is a parameter that seems to influence the prognosis in patients undergoing surgical treatment for NSCLC, especially in the early stages. In particular, some studies have reported that the number of lymph nodes removed not only increases the possibility of upstaging, but also show that the prognosis improves with an increase in the number of lymph nodes removed [30,31].

Results from analyzing this parameter in patients with persistent nodal involvement in our study (N1 + N2) showed that patients with more than six mediastinal lymph nodes presented a better survival rate compared to patients with less than six removed nodes, with the *p*-value close to the statistical significance (*p* = 0.057). Despite the fact it is extremely hard to demonstrate, it is possible that, in these patients, the surgical clearance of the involved lymph nodes may also have ensured a survival benefit. However, the reasons why the lymphadenectomy was incomplete are still not clear and less mediastinal radical dissection should have been considered. In fact, in most cases, this occurred in lymph node tissues with a hard, fibrous consistency, closely adhered to the mediastinal structures for which dissection was difficult and risky, which could lead to the damage of noble structures such as large vessels, trachea and esophagus. Regarding the characteristics reported, it is also difficult to exclude that this picture could also conceal the presence of the persistence of the disease at this level, considering the fact that the resection was incomplete. In spite of the few data available, our results can be comparable with results reported in other literature where surgical risks were linked to lymphadenectomy post-NAD and an extensive variability, considering the number of resected nodes, ranging between 0–16 for N1 and 1–38 for N2 [15,32].

As a result, for patients in whom a radical mediastinal dissection is not technically feasible, it may be appropriate to repeat the performance of a minimally invasive mediastinal staging in the postoperative period, in order to identify a possible residual disease. In this way, it would still be possible to identify patients who could benefit from adjuvant therapies or specific radiotherapy protocols, with better patient selection for postoperative radiotherapy [2,3,8]. 

Despite the good results obtained in this study, there are some limitations, especially with regards to its retrospective nature and its duration. In particular, different neoadjuvant and adjuvant protocols were used over the years, thereby limiting the possibility to analyze the prognostic role of every single protocol. On the other hand, in this monocentric study, all patients underwent multidisciplinary pretreatment discussions, and NAD and adjuvant were administered in accordance with the different guidelines according to the period.

Therefore, we preferred to include all patients that underwent neoadjuvant therapy independently based on the type of protocol and also because the main objective was to consider the prognostic nodal factors in these patients. However, we did not take into account the different NAD protocols because they were difficult to compare. Similarly, we also included pneumonectomy to cover the entire possible surgery spectrum in these kind of patients. Interestingly, pneumonectomy was a negative prognostic factor using univariable analysis only, but we did not obtain 30- or 90-day mortality. It is possible that these results were related to an advanced stage, requiring pneumonectomy and not necessarily the intervention itself, even if it is difficult to demonstrate. 

Another possible limitation is related to the early study period, in which PET and minimally invasive techniques for restaging were not available, thereby affecting the pretreatment staging and clinical or pathological response evaluation [11]. On the other hand, all patients with suspected localization underwent “ad hoc” supplemental staging exams, such as bone scintigraphy or MRI, in case of suspected metastases, and biopsy was always attempted in case of suspected localization. Importantly, the surgical indication was always determined by the multidisciplinary discussion, which may have played a role in improving survival. 

Moreover, the extent of the lymphadenectomy was not homogeneous among patients. However, as reported in the discussion, different NAD-related factors may have influenced its extension and the planned lymphadenectomy consisted of a radical mediastinal dissection. 

Finally, the relatively low number of patients with persistent nodal involvement may have limited the statistical analysis in this subgroup, with comparisons such as pN1 vs. pN2 or #RN < 6 vs. #RN ≥ 6 that presented a large survival difference even if it was not statistically significant. On the other hand, it must be emphasized that this is one of the few and larger studies in the literature that analyzed the nodal parameters in patients with persistent nodal involvement, suggesting further evaluations for the management of this particular class of patients. 

## 5. Conclusions

Our study confirmed the prognostic role of mediastinal downstaging and the importance of adjuvant therapy for survival improvement in patients that underwent multimodal treatment for stage III NSCLC.

In addition, we analyzed the role of the lymph node parameters in patients with persistent nodal involvement and discovered that the lymph node ratio is a promising prognosticator in this particular class of patients.

However, further larger studies are needed to validate these data.

## Figures and Tables

**Table 1:** The inclusion and exclusion criteria for patients that underwent neoadjuvant treatment.

Inclusion Criteria	Exclusion Criteria
Preoperative diagnosis of NSCLC	Bulky nodal involvement
Potentially resectable tumor	Presence of contralateral lymph-nodes metastases
Presence of ipsilateral mediastinal nodal involvement histologically proven	Presence of distant metastases
Age > 18 years	Lack of post-induction staging
Multidisciplinary discussion before treatment	Disease progression during induction therapy
Induction therapy (radiotherapy, chemotherapy or radio-chemotherapy)	
Complete anatomical lung resection (lobectomy, bilobectomy, pneumonectomy)	

**Table 2:** Clinical and pathological characteristics of the entire cohort.

Clinical and Pathological Characteristics	
**Age**	60.7 ± 12
**Sex**	
Male	144 (80%)
Female	36 (20%)
**Neo-adjuvant therapy**	
Chemo-radiotherapy	121 (67.2%)
Chemotherapy	59 (32.8%)
**Surgery**	
Lobectomy/bilobectomy	140 (77.7%)
Pneumonectomy	40 (22.3%)
**Clinical Stage**	
IIIA	148 (82.2%)
IIIB (T3-T4N2)	32 (17.8%)
**Pathological Stage**	
Complete response	46 (25.5%)
I-IIA	65 (36.1%)
IIB	27 (15.0%)
III	42 (23.4%)
**Evaluation post therapy**	
Downstaging	137 (76.1%)
Stable stage	43 (23.9%)
**Histology**	
Adenocarcinoma	88 (48.8%)
Squamous Cell Carcinoma	78 (43.3%)
Adenosquamous	14 (7.9%)
**Adiuvant Therapy**	44 (24.4%)

**Table 3:** Lymph node characteristics after neoadjuvant therapy.

Lymph-Node Characteristics	
**ycN0** **–** **N1**	137 (76.1%)
**ycN2**	43 (23.9%)
**ypN2 downstaging**	152 (84.4%)
**ypN1**	29 (16.1%)
**ypN2**	28 (15.5%)
**# Lymph** **-** **node removed (mean)**	8.5 ± 7.4
**# Lymph** **-** **node involved N2 (mean)**	1.2 ± 2.9
**# mediastinal station (mean)**	2.5 ± 1.3
**Node ratio < 50%**	25 (48.3%)

**Table 4:** Univariable Analysis for overall survival.

	*p*-Value
Age	0.003
Sex	0.470
N2 station previous therapy	0.413
Subcarinal involvement	0.382
Type of therapy	0.903
yc Stage	0.068
ycT	0.343
Type of resection	0.002
pTNM	0.269
Histology	0.178
pT	0.341
pN	0.161
pN2	0.013
Downstaging	0.295
Lymph-node downstaging	0.068
Complete response	0.325
Metastatic lymph-node	0.502
Numer of nodal stations removed	0.209
Number of lymph-nodes removed	0.992
Adjuvant therapy	0.045

**Table 5:** Multivariable analysis. (OS: overall survival, HR: Hazard Ratio, CI: confidence interval).

Variables	Univariable OS	Multivariable OS	
	*p*-Value	HR (CI 95%)	*p*-Value
**Age**	0.003	0.956 (0.90–0.982)	0.002
**Clinical N0-N1 post NAD** Yes vs. No	0.01	-	-
**Lobectomy/bilobectomy** Yes vs. no	0.002	0.620 (0.305–1.262)	0.187
**Pathological N2** Yes vs. no	0.013	2.337 (1.037–5.266)	0.041
**Adjuvant Therapy** No vs. Yes	0.045	2.335 (1.149–4.7461)	0.019

**Table 6:** Univariable analysis in patients with persistent nodal involvement (HR: Hazard Ratio, CI: confidence interval).

Overall Survival		
Variable	*p*-Value	HR (95% CI)
pT	0.795	0.529 (0.63–4.460)
pN (N1 vs. N2)	0.152	0.535 (0.227–1.260)
Number of resected Lymph-nodes (<6 vs. ≥6)	0.068	2.453 (0.936–6.420)
Number of metastatic N1 lymphnodes (1 vs. multiple)	0.701	1.518 (0.564–4.088)
Number of metastatic N2 lymphnodes (1 vs. multiple)	0.837	1.351 (0.491–3-721)
Node Ratio (<50% vs. ≥50%)	**0.036**	0.357 (0.135–0.943)
Number of metastatic lymph-nodes (1 vs. multiple)	0.411	1.816 (0.698–4.730)
Number of removed station N2 <3 vs. >3	0.621	1.263 (0.501–3.186)
Number of metastatic N2 station (single vs. multiple)	0.947	1.182 (0.396–3.531)

## Data Availability

Data are property of the fondazione policlinico Universitario A. Gemelli and may be visualized if needed after authors approval.

## Supplementary Materials

The following supporting information can be downloaded at: https://www.mdpi.com/article/10.3390/life12111753/s1, Supplemental Table S1.
