# Supplementary material for: Clinical Stage III NSCLC Patients Treated with Neoadjuvant Therapy and Surgery: The Prognostic Role of Nodal Characteristics
Source: Life (Basel). 2022 Nov 1;12(11):1753. doi: 10.3390/life12111753 (PMC9692699; doi:10.3390/life12111753)
Supplement: Supplementary file 1 [file life-12-01753-s001.zip › life-1987269-supplementary.pdf]

Supplemental Table S1. Cancer Specific Survival. Univariable and multivariable analysis.

| | Univariable | Multivariable | |
|---|---|---|---|
| | **p-value** | **p-value** | **HR (95%CI)** |
| Age | **<0.001** | **<0.001** | 0.918(0.885-0.953) |
| Sex | 0.726 | | |
| N2 station previous therapy | 0.904 | | |
| Subcarinal involvement | 0.856 | | |
| Type of therapy | 0.637 | | |
| yc Stage | 0.168 | | |
| ycT | 0.318 | | |
| ycN0N1 | **0.035** | **0.004** | 0.286(0.122-0.669) |
| Type of resection | **0.003** | 0.812 | 0.893(0.353-2.263) |
| pTNM | 0.099 | | |
| Histology | 0.132 | | |
| pT | 0.260 | | |
| pN | 0.686 | | |
| pN2 | 0.200 | | |
| Downstaging | 0.713 | | |
| Lymph-node downstaging | 0.539 | | |
| Complete response | 0.119 | | |
| Metastatic lymph-node | 0.494 | | |
| Numer of nodal stations removed | 0.503 | | |
| Number of lymph-nodes removed >6 | 0.134 | | |
| Adiuvant therapy | 0.057 | **0.027** | **2.2628(1.115-6.194)** |
